# Caching for where and what: evidence for a mnemonic strategy in a scatter-hoarder

**DOI:** 10.1098/rsos.170958

**Published:** 2017-09-13

**Authors:** Mikel M. Delgado, Lucia F. Jacobs

**Affiliations:** Department of Psychology, University of California at Berkeley, Berkeley, CA, USA

**Keywords:** chunking, tree squirrels, memory, foraging decisions, food-storing, seed dispersal

## Abstract

Scatter-hoarding animals face the task of maximizing retrieval of their scattered food caches while minimizing loss to pilferers. This demand should select for mnemonics, such as chunking, i.e. a hierarchical cognitive representation that is known to improve recall. Spatial chunking, where caches with the same type of content are related to each other in physical location and memory, would be one such mechanism. Here we tested the hypothesis that scatter-hoarding eastern fox squirrels (*Sciurus niger*) are organizing their caches in spatial patterns consistent with a chunking strategy. We presented 45 individual wild fox squirrels with a series of 16 nuts of four different species, either in runs of four of the same species or 16 nuts offered in a pseudorandom order. Squirrels either collected each nut from a different location or collected all nuts from a single location; we then mapped their subsequent cache distributions using GPS. The chunking hypothesis predicted that squirrels would spatially organize caches by nut species, regardless of presentation order. Our results instead demonstrated that squirrels spatially chunked their caches by nut species but only when caching food that was foraged from a single location. This first demonstration of spatial chunking in a scatter hoarder underscores the cognitive demand of scatter hoarding.

## 1. Introduction

Scatter-hoarding animals face the formidable challenge of creating diverse, ephemeral cache distributions whose location they can remember accurately enough to retrieve later. It has been well-established that scatter-hoarding animals can remember the locations of caches they make (e.g. [1–4]), but they must also remember the contents of a cache, such as a black-capped chickadee (*Poecile atricapillus*) remembering whether a seed is shelled or unshelled [5]. Scatter hoarders, such as the western scrub jay (*Aphelocoma coerulescens*), can also remember when a cache was made, a form of episodic-like memory [6].

Hierarchically organizing caches by content should theoretically improve a scatter hoarder's ability to accurately recall cache locations. This cognitive process is known as chunking, where a chunk is a collection of items that have commonalities and discriminability from other chunks [7]. Spatial chunking has been already demonstrated to improve spatial recall in laboratory rats (*Rattus norvegicus*) retrieving three types of food rewards in a 12-arm radial maze. When food items of a certain type were consistently found in the same locations, the rats retrieved the rewards in order of food preference [8,9], similar to the behaviour of the black-capped chickadees [5]. The rats also retrieved preferred favoured food items with fewer arm visits under these conditions [8]. If item type was switched but chunk integrity was maintained (i.e. food A was replaced with food B), rats retrieved preferred items in fewer visits compared to their performance when food type was randomly redistributed [9]. Such a hierarchical memory representation has also been demonstrated in songbirds in their organization of song syllables to be learned (e.g. [10]).

We tested this hypothesis in wild fox squirrels, who cache and consume the large seeds of several tree species in their native eastern deciduous forest [11]. Fox squirrels and closely related eastern grey squirrels (*S. carolinensis*) respond adaptively to each nut that is encountered, adjusting eating and cache decisions to its relative value, the abundance of food, the type of food, and the perception of risk of pilfering [1,12–14]. Squirrels cache preferred foods farther from the source (e.g. [13,14]) and at lower densities [15]. Finally, squirrels have been shown to encode and recall the spatial locations of their caches [16,17].

We tested the hypothesis that a scatter-hoarding fox squirrel employs a chunking strategy by allowing them to collect a series of four species of tree seeds, where both the location of the food source and the serial order of the nuts collected was systematically varied. We varied the complexity of the series to increase the cognitive load and measured the spatial overlap between caches of different nut species. We defined chunking as the creation of exclusive nut-species cache distributions that could not be explained by any other heuristic and predicted that chunking would vary with cognitive load.

## 2. Material and methods

The study was conducted on the University of California at Berkeley campus, with a population of marked, free-ranging fox squirrels, as described in earlier studies [12,13]. Forty-five individual squirrels (17 female and 28 male) participated in the study.

We collected data from 10.00 to 16.00 between June 2012 and April 2014, avoiding the fall season when food is abundant due to tree mast. During the fall, squirrel caching behaviour is most stereotyped, and least variable [13]. For the eleven squirrels who participated in multiple sessions, the order of condition was randomized and predetermined, and at least one week passed between sessions.

During each session, a squirrel was given a series of 16 individual nuts, in the shell, of four species that vary in weight, size and nutritional content [18]: almonds (*Prunus dulcis*, designated as A); hazelnuts (*Corylus americana*, H); pecans (*Carya illinoinensis*, P); and walnuts (*Juglans regia*, W). Previous studies demonstrated that squirrels show differential responding when caching almonds, hazelnuts and walnuts in comparison to other nut species, and in general show the ability to discriminate between different nut qualities, such as weight and perishability [12,13,19,20]. Each nut was weighed and assigned a unique code in order to include weight in analyses.

Squirrels were given one nut per trial, either in runs of four (RUNS; four nuts of same species, e.g. AAAAHHHHPPPPWWWW) or in a pseudorandom order (PSEUDO; 16 nuts, no species was given twice in a row, e.g. AWHPWHAPHWPAWHPA). If a squirrel ate a nut instead of caching, the nut was replaced with one of the same type until the squirrel cached again. Squirrels sourced nuts under one of two spatial conditions: Multiple Locations (MULTI), where the squirrel would be given the next nut in the location it had just cached, and Central Location (CEN), where the squirrel had to return to a single location to collect the next nut.

One experimenter served as the feeder, offering the squirrel each nut in the sequence. A second experimenter recorded the starting location using a handheld GPS navigator (Garmin Etrex H or 10). A third experimenter recorded the location of the cache, which in the MULTI source was also the next starting location. The first experimenter gave the squirrel the next nut. The second and third experimenters then alternated recording cache locations, to allow for an adequate number of GPS waypoints to be recorded for each cache. To minimize observer effects, experimenters maintained a distance of 5–10 m from the squirrel. There were 58 sessions (RUNS-MULTI: *N* = 14 (6 female, 8 male); PSEUDO-MULTI: *N* = 14 (4 female, 10 male); RUNS-CEN: *N* = 15 (6 female, 9 male); PSEUDO-CEN: *N* = 15 (8 female, 7 male)). Three squirrels did not cache all 16 nuts: one cached 11 nuts, one cached 12 nuts, and one cached 14 out of 16 nuts.

## 3. Data analysis

Least-squares mixed models and MANOVA tests were analysed with JMP Pro12.0 (SAS, Cary, NC, USA). We included the factors Order (Runs/Pseudorandom) and Source (Multiple/Central), as well as their interaction. We also included sex as an independent variable, and squirrel identity as a random effect in all models to account for individual variability and repeated measures. As weight and nut type were highly correlated, with all nut types having significantly different weights from each other (*F*_3,826.9_ = 4789.05, *p* < 0.001), weight was not included in the models. Follow up pairwise comparisons were conducted using Tukey's HSD.

Geographical data were analysed using ArcGIS version 10.3 (ESRI, Redlands, CA, USA). Based on error between known distances and distances calculated by GPS receivers for 75 locations (*x* = 1.3 m, 95% CI (1.04, 1.65)), we created a buffer (1.65 m) around each waypoint that likely held the true cache. Four polygons were created including the waypoints for each squirrel's caches by nut species and alternately by sequence (the first four caches, second four caches, and so forth). We calculated the intersection of these polygons, measuring areas where there was no overlap, or overlap between two, three or four nut types or sequences of four caches. Overlap was assessed as per cent of each squirrel's total cache area during a session.

## 4. Results

**Figure 1.**
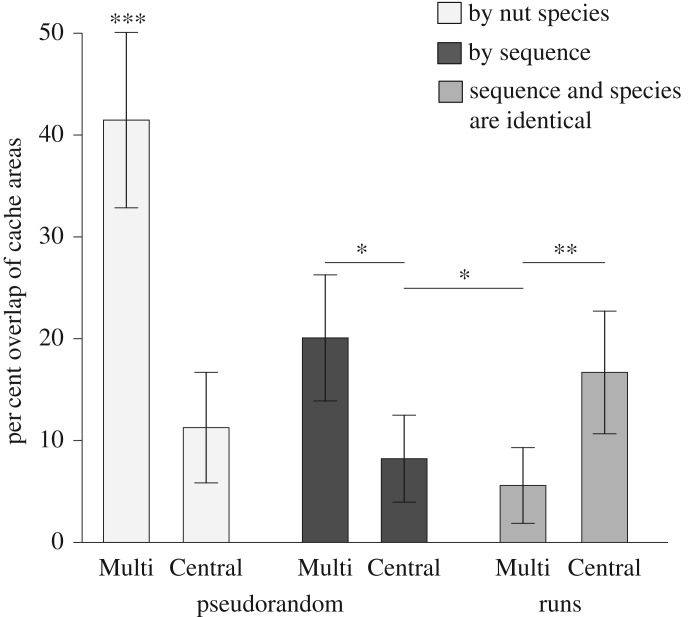
Means and 95% CIs for per cent of overlap by cache species or sequential groups of four (i.e. nuts 1–4; nuts 5–8; nuts 9–12; nuts 13–16) for each condition. Squirrels minimize overlap between nut species when central foraging regardless of order of presentation. Asterisks show statistical differences between cache types: *p < 0.05, **p < 0.001, ***statistically different from all other categories.

**Figure 2.**
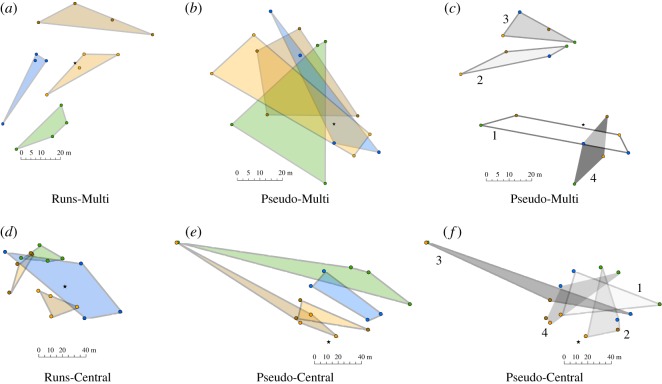
Examples of how squirrels distributed their caches by nut type and sequential order under different experimental conditions. Top row: Caches made by one adult female fox squirrel in the conditions RUNS-MULTI (*a*) and PSEUDO-MULTI (*b*,*c*); (*b*) and (*c*) show the same data analysed by Species versus Sequence. The low overlap between caches of different nut species can be explained by avoiding overlap with previous caches. (*d*,*e*,*f*) Caches made by one adult male squirrel in conditions RUNS-CEN (*d*) and PSEUDO-CEN (*e*,*f*); (*e*) and (*f*) show the same data analysed by Species versus Sequence. Squirrels in these conditions organized caches by nut species rather than sequence. Numbers indicate the caching order in sequential runs of four. Nut species: orange = almonds, blue = hazelnuts, green = pecans, brown = walnuts. Star indicates session starting points.

Order (*F*_1,31.97_ = 29.82, *p* < 0.001, *r* = 0.69), Source (*F*_1,50.25_ = 8.34, *p* = 0.006, *r* = 0.38) and their interaction (*F*_1,35.1_ = 53.02, *p* < 0.001, *r* = 0.78) were all related to how much squirrels overlapped their caches by nut type. Squirrels overlapped caches by nut type more in condition PSEUDO-MULTI (figures 1 and 2). There was no effect of sex (*p* = 0.72).

Because the overlap effect could also arise from other cache heuristics, such as a squirrel sequentially using different locales for the next few caches, we also examined how much squirrels overlapped their caches by sequence rather than by species. Squirrels overlapped caches less by sequence when foraging in MULTI (*F*_1,48.51_ = 19.90, *p* < 0.001, *r* = 0.54; figures 1 and 2). There was no effect of Order, and no interaction effect between Source and Order.

We compared the differences between overlap by nut species or sequence by Source for Pseudorandom data only. Results suggested an effect of species versus sequence, by Source, and their interaction (*F*_3,24_ = 25.62, *p* < 0.001). Squirrels overlapped caches more in the Multiple Source condition than Central Source (*t*_54_ = 3.13, *p* = 0.003); and more by Species than Sequence (*t*_54_ = −4.17, *p* < 0.001).

We compared the difference between the Central and Multiple Sourced conditions when squirrels received nuts in Runs. By necessity, the data for nuts and sequence were the same. A non-directional *t*-test showed more cache overlap when squirrels received nuts from a Central location (*t*_23_ = −3.37, *p* = 0.003). See figures 1 and 2.

Finally, we also tested whether this separation of caches by nut when central foraging could be achieved by a simpler heuristic, such as adjusting distance travelled from the food source based on nut size. For example, squirrels could be differentiating between the large nut species (pecans, average weight, *x* = 8.16 g; walnuts, *x* = 11.75 g) and the smaller nut species (hazelnuts, *x* = 3.22 g; almonds, *x* = 3.44 g). We found that the distributions of large nuts overlapped with each other more than the distributions of small nuts (52.33% versus 11.59%) in order RUNS (*F*_1,27_ = 5.26, *p* = 0.030, *r* = 0.38). In order PSEUDO, the overlap between large nuts (23.1%) and small nuts (21.29%) was similar.

## 5. Discussion

The present study provides the first evidence that a scatter hoarder could employ spatial chunking during cache distribution as a cognitive strategy to decrease memory load and hence increase accuracy of retrieval. When foraging from a central location, squirrels showed little overlap of caches by nut species, regardless of the order in which different food types were presented (figure 2*d*,*e*). Squirrels may thus be able to organize caches hierarchically by cache contents, i.e. spatially chunk cache locations, regardless of the order in which they have encountered different nut species under the natural conditions of a species-diverse deciduous forest.

When squirrels were encountering runs of nut types from multiple locations (RUNS-MULTI), they never overlapped the caches of more than two species of nuts (figure 2*a*). Instead, in PSEUDO-MULTI, which we anticipated would place the largest burden on memory, squirrels overlapped the caches of at least three nut species (figure 2*b*). When food was sourced from multiple locations, squirrels appeared to be using a different heuristic, caching to prevent overlap with areas they had previously cached in during that session rather than organizing caches by nut species (figure 2*a*,*c*). These observations suggest that when lacking the cognitive anchor of a central food source, fox squirrels use a different and perhaps simpler heuristic, to simply avoid the areas where they had previously cached. Another explanation for the break-down of chunking in this condition is not a constraint on memory capacity but instead an adaptive response to the higher energetic costs of spatial chunking when similar items are farther apart in space and time. This is because when encountering nuts pseudo-randomly, from multiple locations, the different items of one species might be more spatially dispersed than the same items encountered at a central source or encountered in runs.

Because seed-bearing trees provide a superabundant food source, a caching squirrel that discovers this source may treat the chosen tree as a central place forager would, bringing seeds in a preferred direction towards the centre of its home range. When eating, squirrels often travel some distance from the food source, particularly in the face of competition [13,21,22]. But whether eating or caching, the distance travelled away from the food source will be influenced by the opportunity cost of leaving an ephemeral food patch to one's competitors for a long period of time.

If the distance travelled is significant, returning to a central food source could be costlier than foraging from a new food source near where the squirrel just cached or ate. Thus, a tree squirrel may face a constant series of decisions – whether to forage from one site or many, similar to the multiple location foraging condition in our study. Our results may reflect the effects of these kinds of foraging decisions and show that squirrels may adjust behaviours dependent on how food is acquired.

Finally, squirrels may be chunking by item value, and this value may be derived from other factors as well as nut species, such as the weight of that individual nut. Other studies have shown that squirrels modulate the distance they will carry a nut of a certain species, carrying larger individual nuts significantly farther than smaller individual nuts of the same species (e.g. [20,23]). Our observation that squirrels, under certain conditions, categorize and respond to a nut as either large or small, also suggests that they could be organizing caches by even more subtle hierarchical structures than simply nut species.

## Permission to carry out fieldwork

This research was conducted under a scientific collecting permit issued by the California Department of Fish and Wildlife.

## Data accessibility

The data from this study can be accessed at doi:10.6084/m9.figshare.5285635.
